# Studying Sub-Dendrograms of Resting-State Functional Networks with Voxel-Wise Hierarchical Clustering

**DOI:** 10.3389/fnhum.2016.00075

**Published:** 2016-03-08

**Authors:** Yanlu Wang, Mussie Msghina, Tie-Qiang Li

**Affiliations:** ^1^Department of Clinical Science, Intervention, and Technology, Karolinska InstituteStockholm, Sweden; ^2^Department of Clinical Neuroscience, Karolinska University HospitalHuddinge, Sweden; ^3^Department of Medical Physics, Karolinska University HospitalHuddinge, Sweden

**Keywords:** hierarchical clustering, resting-state networks, intra-network connectivity, somatosensory network, visual network, resting-state fMRI

## Abstract

Hierarchical clustering is a useful data-driven approach to classify complex data and has been used to analyze resting-state functional magnetic resonance imaging (fMRI) data and derive functional networks of the human brain at very large scale, such as the entire visual or sensory-motor cortex. In this study, we developed a voxel-wise, whole-brain hierarchical clustering framework to perform multi-stage analysis of group-averaged resting-state fMRI data in different levels of detail. With the framework we analyzed particularly the somatosensory motor and visual systems in fine details and constructed the corresponding sub-dendrograms, which corroborate consistently with the known modular organizations from previous clinical and experimental studies. The framework provides a useful tool for data-driven analysis of resting-state fMRI data to gain insight into the hierarchical organization and degree of functional modulation among the sub-units.

## Introduction

Different clustering techniques have been used for exploratory analysis of resting-state functional magnetic resonance imaging (fMRI) data aimed to group together functionally similar voxels or regions of interests (ROIs) and identify functionally connected brain networks. These include, among others, fuzzy C-means (Baumgartner et al., 1997; Hilgetag et al., 2000), spectral clustering (Snyder et al., 1997; Mattingley et al., 1998), K-means clustering (Fogassi et al., 2005), hierarchical clustering (Cordes et al., 2002; Foxe et al., 2002; Menon and Uddin, 2010; Wang and Li, 2013), consensus clustering (Moretti and Muñoz, 2013), and constrained clustering (Snyder et al., 1997; Foxe et al., 2002). Hierarchical clustering analysis (HCA) has not been used as fluently as other clustering methods in the analysis of resting-state fMRI data probably due to its poor scalability, high complexity and sensitivity to noise outliers. However, HCA is completely deterministic and can stratify inherently the data into a hierarchical structure (Zhou et al., 2006; Marrelec et al., 2008; Gómez-Laberge et al., 2011; Boly et al., 2012). Although the structure nature of resting-state functional networks (RFNs) is still a matter of debate (Sporns, 2014), the notion that both structure and function connection networks exhibit a hierarchical organization of distinct brain modules that communicate through connector hubs is supported by a massive body of evidence ranging from cellular circuit of neuron connections to large-scale brain networks (Hilgetag et al., 2000; Zhou et al., 2006; Cohen et al., 2008; Ferrarini et al., 2009; Park and Friston, 2013; Zhen et al., 2013; Russo et al., 2014). Hence, extraction and characterization of such a hierarchical organization is an important issue in the study of brain function networks.

In spite of the successful application of HCA in extracting RFNs (Liu et al., 2012; Wang and Li, 2013; Alho et al., 2014), the results are strongly affected by the specified number of network clusters, which is not known *a priori*. Almost all of the previous clustering studies of resting-state fMRI attempt to parcel the data into a predefined number of clusters so that the clusters have high intra-cluster similarity and low inter-cluster similarity according to a chosen distance metric. Therefore, the outcome depends significantly on the choice of distance metric and the predefined number of clusters. In HCA, the cutting depth of dendrogram directly defines the number of produced clusters. Typically, over 10 RFNs are extracted from the group-averaged resting-state fMRI data (Cavanna and Trimble, 2006; Bellec et al., 2010; Wang and Li, 2013) in reference to the result from independent component analysis (ICA) studies (Damoiseaux et al., 2006; Smith et al., 2009), However, ICA result itself is also plagued with the issue of unknown number of independent components (Meunier et al., 2010; Bullmore and Sporns, 2012).

Due to lack of “ground truth” for the number of RFNs, clustering studies of resting-state fMRI data have to deal with the optimization of the number of clusters and assessment of clustering quality. Cluster validity index provides a tool to evaluate the performance of clustering algorithm. Many different cluster validity indexes have been proposed in the literature, e.g., Milligan and Cooper (Milligan and Cooper, 1985) presented a comparison of 30 HCA validity indexes. In general, these indices can be classified as internal and external indexes, the former are usually based on information intrinsic to the data, while the latter are based on prior knowledge about the data. The optimal number of clusters can be determined by identifying the “knee point” (where the validity index exhibits a sharp change) among the validity index values as a function of different numbers of clusters. This procedure can fail in practice, because there might not exist any “knee points” or the existence of multiple. Therefore, knowing how to define a robust clustering criterion is critical and requires a good understanding of the data.

The main purpose of this study is to use cluster size as validity criteria for the investigation of the intra-network hierarchical organization of functional networks based on group-averaged resting-state fMRI data. Our approach is based on multi-stage dissection of the full connectivity dendrogram derived using the time course of individual voxel (Wang and Li, 2013). We employed an average-linking agglomerative hierarchical clustering algorithm to generate the full correlation-coefficient dendrogram for gray matter in the brain with over 13 × 10^3^ nodes. We developed a framework that can be used to retrieve the entire linkage tree, dissect it at any desired level and track a given mother node and its associated children clusters in any two consecutive cuts of the dendrogram. Based on stop criteria derived from the internal characteristics of the produced clusters, such as cluster size, weakest linkage, and inconsistency coefficient (IC), we devised an iterative procedure to extract the potential functional networks and sub-networks within a given functional network. Particularly, we investigated the somatosensory motor (SSM) and visual systems in fine details and constructed their sub-dendrograms according to the tracked hierarchical association and distance metrics among the sub-units.

## Materials and methods

### Ethics statement

The Central Ethical Review Board in Stockholm region approved this study permission including the recruiting ad and consent form used to provide information and obtain consent. All participants provided informed consent by voluntary signature.

### Data acquisition

Resting-state fMRI measurements were conducted for a total of 84 normal adult subjects (male = 40, 46, age = 21–84) using a 3T whole-body clinical MRI scanner (TIM Trio, Siemens Healthcare, Erlangen, Germany). A single-shot 2D gradient-recalled echo echo-planar imaging sequence was used with the following acquisition parameters: 32 transverse slices (3.6 mm thickness), TR/TE = 2000/35 ms, FOV = 220 mm, matrix size = 64 × 64, flip angle = 90°, 300 dynamic timeframes, IPAT = 2. A 32-channel phased-array head coil was used for the signal reception. Foam paddings were used to for every subject reduce the head motions.

### Preprocessing

The resting-state fMRI datasets underwent the same preprocessing procedure, which were performed with AFNI (http://afni.nimh.nih.gov/afni) and FSL (http://www.fmrib.ox.ac.uk/fsl) programs with a bash wrapper shell. The first 10 time frames in each data set were removed to ensure signal steady state. After temporal de-spiking, six-parameter rigid body image registration was performed for motion correction. The average volume for each motion-corrected time series was used to generate a brain mask to minimize the inclusion of the extra-cerebral tissues. Spatial normalization to the standard Talairach template was performed using a 12-parameter affine transformation and mutual-information cost function. The data was then resampled to isotropic resolution using a Gaussian kernel with FWHM = 4 mm. Nuisance signal removal was achieved by voxel-wise regression using the 14 regressors based on the motion correction parameters, average signal of the ventricles and their 1st order derivatives. To avoid creating excessive negative functional connectivity no regression of white matter signal was included. After baseline trend removal up to the third order polynomial, effective band-pass filtering was performed using low-pass filtering at 0.08 Hz. Local Gaussian smoothing up to FWHM = 6 mm was performed using an eroded gray matter mask to reduce unnecessary partial volume effect from CSF and white matter. The actual FWHM of the smoothed data was 6.2 ± 2 mm as estimated by using the AFNI program, 3dFWHM.

### Hierarchical clustering to extract RFNs

Clustering was restricted to voxels inside the gray-matter using a gray-matter masked derived from FSL gray-matter tissue priors (http://www.fmrib.ox.ac.uk/fsl). For each subject, the Pearson's cross correlation (CC) distances were calculated voxel-wise for all datasets. The correlation distances were then evaluated with a threshold ≤ 0.7 corresponding to a correlation coefficient threshold CC ≥ 0.3 (Cordes et al., 2002). After thresholding, ~1.1% of the correlation coefficients remained. Cordes et al. (2002) used previously the same CC threshold of 0.3 for voxel-based hierarchical clustering and the same threshold was opted here for the individual dataset. We systematically changed the CC threshold and found that increasing the threshold above 0.3 resulted in the loss of robustness of the algorithm. A threshold of CC ≥ 0.4 resulted in only about 0.1% of the values remained. After thresholding, all values above the threshold were set to an arbitrary large value. The cross correlation matrices for all subjects were then averaged together voxel-wise. The averaged distance matrix was then computed, which was used to perform hierarchical clustering through an average-linking agglomerative clustering algorithm as the basis for the framework (Wang and Li, 2013).

A brief description of the algorithm is summarized as follows: Given a set of *N* voxels to be clustered, and a corresponding *N* × *N* distance matrix: (1) Assign each voxel to a cluster, resulting in *N* clusters, with each cluster containing just one voxel. The distances between the clusters are the distances among the voxels; (2) Find the closest pair of clusters; (3) Merge the closest pair of clusters, resulting in one cluster less in total; (4) Repeat 2–3 until only a single cluster remains. Step 3 can be performed in a variety of ways, referred to as linkage methods. The type of linkage in a hierarchical clustering algorithm refers to how the algorithm determines distance between newly formed clusters to all other voxels and clusters. Single-linkage takes the shortest distance between new clusters against the rest of the data, maximum-linkage takes the longest distance, and average-linkage takes the average. In our application, voxels within a cluster corresponding to a functional connectivity network should be highly correlated to each other. Hence, single-linkage is not desirable in this application. Maximum-linkage forces the algorithm to solely determine clusters with all voxels having high correlations to each other without exceptions. Average-linkage relaxes somewhat the intra-cluster connectivity requirements compared to maximum-linkage by taking the average distance. Hence, average-linkage was opted to take into account of the potential noise residues. We have previously described the algorithm in more details elsewhere (Wang and Li, 2013).

The algorithm produces a binary tree, known as a dendrogram, presenting the hierarchical organization of the individual elements as leafs according the pre-defined distance measure. *k* number of clusters can be retrieved by cutting the *k-1* longest links in the dendrogram. Since the number of meaningful clusters is unknown *a priori* and is affected by the noise level of the resting-state fMRI data, it is difficult to use the number of clusters as the termination criteria. Through tests on different cutting depths of the dendrogram, we developed an iterative scheme with termination criteria based on cluster size (*S*) to extract RFNs. A cluster larger than 5000 voxels in size (*S* ≥ 5000) is considered too large to be a single RFNs, because the whole-brain gray matter mask that was used has 13,312 voxels and analysis of the ICA results showed that clusters with more than 5000 voxels are usually too large to be considered as a single coherent RFN and should be refined further. Therefore, we choose 5000 as the upper limit for a cluster to be considered as an independent RFN. Analyses of the resulted clusters at different cutting levels showed that many of clusters are small clusters with < 50 voxels (Wang and Li, 2013) and are not associated with any known RFNs. Therefore, we choose 50 as the lower limit for clusters to be considered as potential RFNs. In order to identify potential RFNs, Clusters with adequate voxel size (50 ≤ *S* ≤ 5000) were carefully examined by comparing their spatial distribution patterns with previously published RFNs in the literatures.

Preliminary analyses of the whole dendrogram indicated that we couldn't extract all known RFNs by a single cut. Therefore, we implemented the following iterative procedure. In the first iteration the cluster count for whole-brain dendrogram (*k*_1_), was set to 64, because using higher cluster counts, such as *k*_*I*_ = 128, did not split the largest cluster instead of giving rise to further division of the smaller clusters. Following the first iteration, clusters above 5000 voxels in size (*S*) were then dissected with a reduced cluster count by a factor of 2 to avoid generating spurious amounts of small clusters, considering the fact that the largest cluster was about half in size compared with the total number of nodes in the whole-brain. In order to identify potential RFNs, the empirical cluster size criteria (50 ≤ *S* ≤ 5000) discussed above was adopted to filter away small clusters (Wang and Li, 2013). The remaining prospective clusters were then carefully examined by comparing their spatial distribution patterns with ICA results and previously published RFNs in the literature (Wang and Li, 2013). Among the identified potential RFN clusters, we selected the RFNs corresponding to the SSM and visual systems for further analysis with the method described below. The basic information for the extracted SSM and visual RFNs is replicated and summarized in Table 1.

**Table 1:** **Summary of the SSM and visual RFNs as extracted by voxel-wise hierarchical clustering of the mean cross-correlation confident matrix derived from the resting-state fMRI data of 86 normal adult subjects**.

**RFN**	**SSM**	**Visual**
Size (Voxels)	1540	1619
CC_min_	0.19	0.18
CC_avg_	0.27	0.29
CC_max_	0.35	0.36
IC_MEAN_	3.10 ± 0.17	3.25 ± 0.34
Divisible nodes	20	19
Sub-networks	14	15

The dissection was terminated at the inconsistency coefficient of 2.65, which corresponds to the first crossing point of the inconsistency coefficient curves for the SSM and visual RFNs.

### Hierarchical modular decomposition of the SSM and visual RFNs

**Figure 1:**
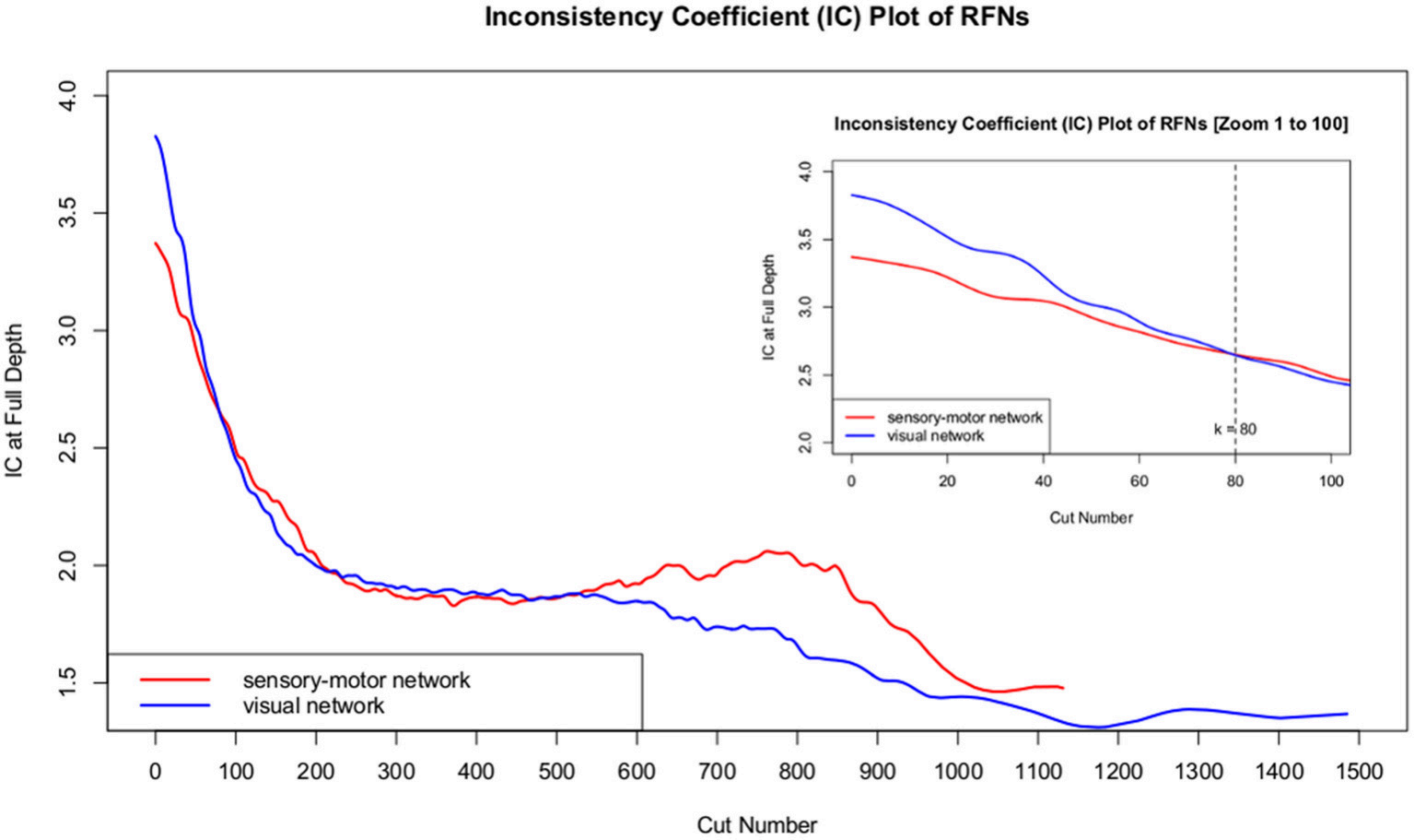
**Inconsistency coefficients at full depth for all nodes in the SSM and visual RFNs**.

One of the outputs from the framework is the whole dendrogram as a simple ASCII file that contains a list of nodes, their linking distance; connections to their children clusters; and the size of children clusters. With the node list and the associated information, any given node, cluster, or sub-tree can be efficiently retrieved. Our framework contains a module that can be used to identify and visualize the split children clusters from any given mother node. For a given RFN (a sub-dendrogram), the module can be used to iterate through the list of nodes ordered in descending heights and systematically dissect through all nodes in the network. For a cut at node *k* > 0, we compare the clusters with the results from the previous cut at node *k*−*1* to identify which cluster is split. We define an extracted cluster as a significant cluster, if its size and minimum inter-voxel correlation coefficient among the voxels within the cluster are sufficiently large to guarantee that the inter-voxel correlation is statistically significant with a family-wise error rate (FWER) *p* ≤ 0.01. Whenever a significant cluster is split into two significant clusters, the mother node is denoted as a dissociable node and its children clusters are labeled for inspection and further analysis. The above process is repeated again for each significant child cluster. In principle, the iteration process can continue down to any desirable level of fine details. To facilitate the comparison between the results from the SSM and visual systems, we choose the cluster count corresponding to the first intersect point of the inconsistency coefficient (IC) curves. The IC value for each non-leaf node in a hierarchical cluster tree is evaluated as the difference in height between the current link and the average height of other links at the two levels adjacent to it normalized by their standard deviation. IC is a measure of separation between the two clusters whose merge is represented by the node in question, compared to the separation between sub-clusters merged within those clusters. The higher the IC value, the less similar the sub-clusters connected by the link. As shown in Figure 1, the IC curves intersect at IC = 2.65 and the corresponding cut level is 80, which is a manageable size both for cluster characterization and visualization. For each RFN, the extracted functional connectivity sub-networks together with their node heights were then used to construct the intra-network sub-dendrogram.

### Statistical assessment of the clustering results

**Figure 2:**
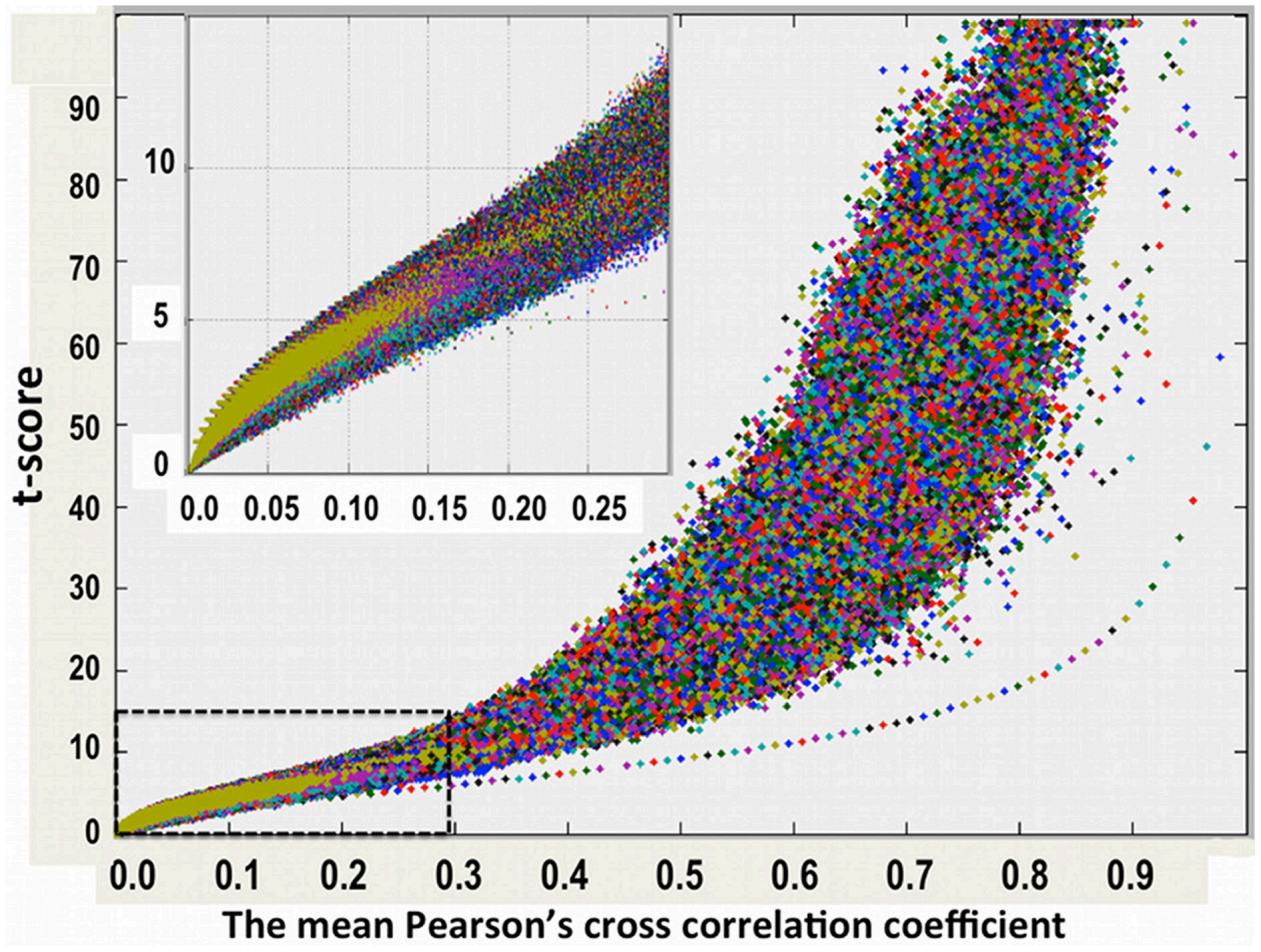
**One-sampled *t*-test score as a function of the cross-correlation coefficients of the resting-state fMRI time courses between voxels inside the brain**. The average results for 84 normal adult volunteers are displayed.

As described above, for each resting-state fMRI dataset, we computed voxel-wise the Pearson's cross-correlation coefficients (CC) and thresholded at CC ≥ 0.3 (corresponding to a distance threshold ≤ 0.7). To assess the statistical significance of the inter-subject averaged CC matrix, we performed voxel-wise one-sampled *t*-test for the thresholded CC data (*N* = 84 subjects) to test the null hypothesis that the inter-subject averaged CC at a given voxel is not significantly different from 0. We computed also the voxel-wise skewness and kurtosis for the inter-subject averaged CC matrix. Examinations of the skewness and kurtosis vs. the mean CC values show that the −1.01 < skewness < −1.0 and 1.0 < kurtosis < 1.02 when the averaged CC ≥ 0.18, indicating that the mean CC can be reasonably approximated as a normal distribution. Figure 2 depicts the scatter plot of the voxel-wise *t*-scores vs. the mean CC values.

**Figure 3:**
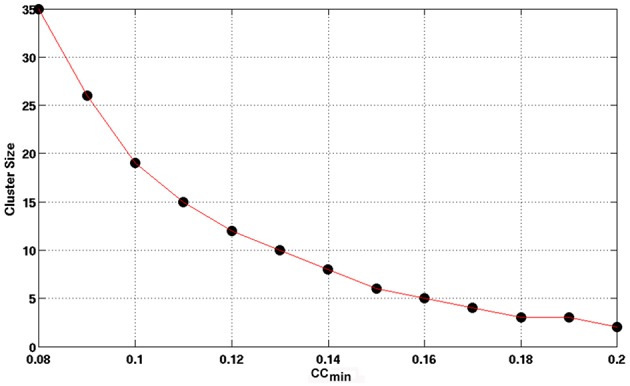
**The minimum cluster size to guarantee a FWER, *p* ≤ 0.01 as a function of the minimum CC value of the cluster**. Clusters with cluster size and minimum CC values above the curve are therefore, statistically significance with FWER, *p* ≤ 0.01 as assessed by Monte Carlo simulation with AlphaSim.

Using the lower boundary of this scattered plot, we obtained the upper limit of the voxel-wise *p*-value corresponding to the minimum CC. We used the AFNI program, AlphaSim, to estimate the minimum cluster size to guarantee a FWER, *p* < 0.01 for a given voxel-wise *p*-value. We employed the following input parameters for the AlphaSim-based simulations: the brain mask, the upper limit of voxel-wise *p*-value corresponding to the minimum CC, FWHM = 6.2 mm, and simulation iterations = 10^6^. FWHM = 6.2 mm was the estimated average by applying the AFNI program, 3dFWHMx, to the final smoothed fMRI data, which was quite close to FWHM = 6 mm used in the final smoothing procedure described above. Figure 3 shows the derived criterion of cluster size as a function of the minimum CC to achieve a FWER, *p* < 0.01 relevant for assessing the HCA clustering result of the average CC matrix of the entire dataset. To gain insight into the reproducibility of the HCA results of averaged resting-state fMRI data, we split the entire dataset into two subsets with the same number of samples in a pseudorandom fashion and performed similar statistical assessment as for the full dataset.

## Results

### Sub-dendrogram of the SSM network

**Figure 4:**
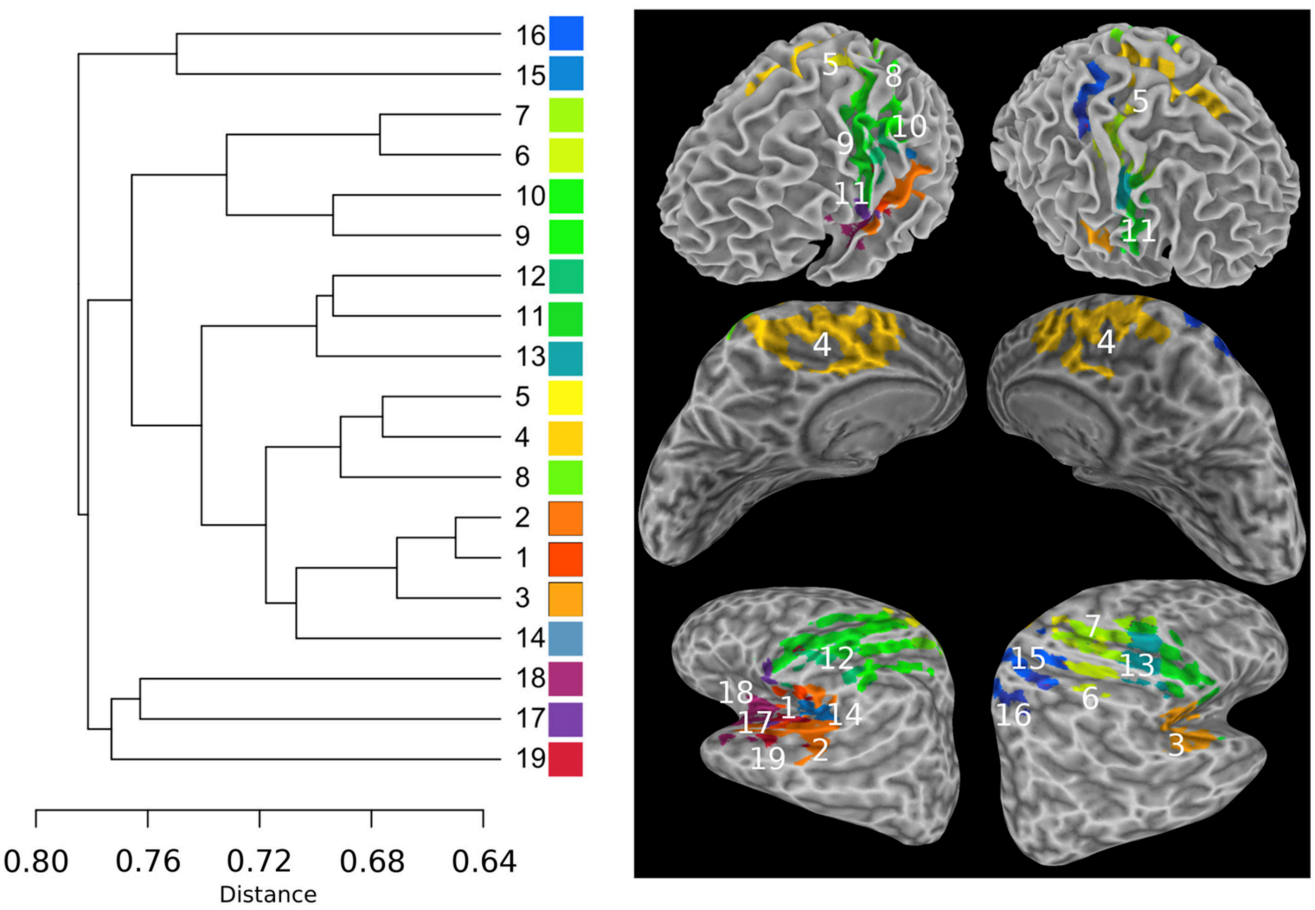
**The intra-network hierarchical organization of the sub-networks extracted from the SSM RFN**. The lines in the sub-dendrogram are drawn in proportion to the distance measure of the nodes (left). The sub-networks are labeled in ascending order according to their distances in the sub-dendrogram. Each sub-network is color coded to depict the neuroanatomical location in the Talairach template. The top panel shows the color-coded sub-networks imposed on the smoothed white matter surface. The middle and bottom panels show the medial and lateral views of the inflated hemispheres, respectively.

**Figure 5:**
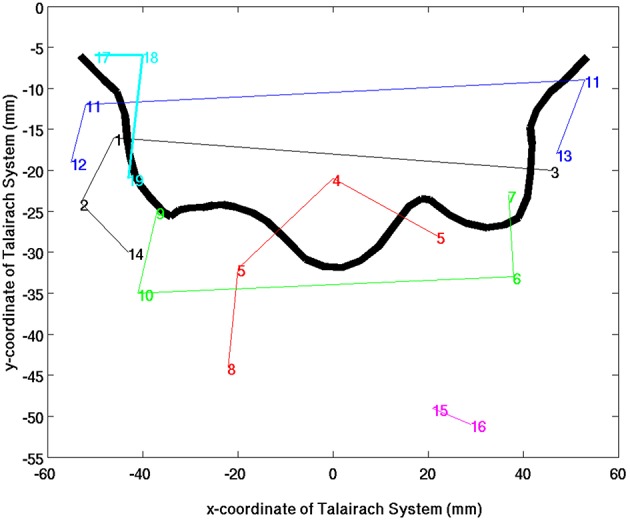
**An overview of SSM sub-network groups in relation to the fontal edge of the central sulcus as a 2D-projection in the Talairach coordinate system**. The colors indicate the different sub-network groups and the lines were drawn to guide the eyes.

The Sub-networks for the SSM RFN were dissected down to 80 nodes (cut level *k* = 79). Among the 80 nodes, 19 sub-networks amongst 20 dissociable nodes were identified (Tables 1, 2) to be statistically significant (*p* < 0.01 and *s*≥12) and functionally distinct. The intra-network hierarchy of the extracted sub-networks and their neuroanatomical locations are depicted in Figure 4. As shown, the extracted sub-networks for the SSM network include the following four groups of bilateral sub-networks: insula-auditory sub-networks (Figure 4, clusters 1–3), paracentral lobule, and cingulate motor cortex sub-networks (Figure 4, clusters 4, 5), sub-networks for facial expression control (Figure 4, clusters 11–13), and sub-networks for hand movement control (Figure 4, clusters 6, 7, 9, and 10). Furthermore, there exist also two groups of unilateral sub-networks: the right parietal sub-network group (Figure 4, clusters 15, 16) and the left insular-STG (superior temporal gyrus) sub-network group (Figure 4, clusters 17–19). An overview of these sub-network groups in relation to the central sulcus is outlined in Figure 5 as a 2D-projection in the Talairach coordinates. This intra-network organization of intrinsic functional connectivity derived from spontaneous activity of the brain at rest reflects consistently the functional and neural anatomic connectivity topography of the SSM network. SSM system in the human brain consists of S1, M1, and some pre/post- central gyrus areas divided into dorsal and ventral subgroups in addition to the parietal operculum and the auditory cortex (Power et al., 2011).

**Table 2:** **Summary of the significant sub-networks extracted from the SSM RFN**.

**Cluster**	**Size**	**Height**	**Cortical location**	**Focal point**
1	25	0.66	Left insula; BA13	(−47, −14, 12)
2	99	0.66	Left Superior Temporal Gyrus (STG); BA41	(−53, −24, 8)
3	99	0.69	Right STG; BA42	(46, −20, 12)
4	370	0.69	Paracentral lobule; BA31; Cingulate−motor	(0, −20, 51)
5	34	0.69	Left postcentral gyrus; BA3	(−20, −32, 60)
			Right postcentral gyrus; BA3	(22, −28, 60)
6	40	0.69	Right Inferior parietal lobule; BA40	(38, −33, 46)
7	55	0.69	Right pre− postostcentral Gyrus; BA3−4	(37, −23, 50)
8	36	0.71	Left Superior Parietal Lobule; BA7	(−22, −44, 57)
9	67	0.71	Left pre− postcentral Gyrus; BA3−4	(−38, −28, 49)
10	63	0.71	Left Inferior parietal lobule; BA40	(−41, −37, 45)
11	150	0.71	Left precentral gyrus; BA4	(−48, −12, 36)
			Right precentral gyrus; BA4	(53, −9, 27)
12	36	0.71	Left postcentral Gyrus; BA2	(−52, −19, 31)
13	32	0.72	Right postcentral Gyrus; BA2	(47, −18, 36)
14	37	0.72	Left STG; BA41	(−39, −30, 16)
15	55	0.77	Right Inferior parietal lobule; BA40	(21, −49, 56)
16	33	0.77	Right Precuneus; BA7	(29, −51, 49)
17	22	0.78	Left STG BA22, Precentral gyrus; BA6	(−50, −6, 6)
18	54	0.78	Left Insula; BA13	(−40, −6, −2)
19	29	0.79	Left STG; BA22; Insula; BA13	(−43, −21, 5)

The sub-networks are labeled in ascending order according to their heights in the hierarchical sub-dendrogram. The closest matching Brodmann area (BA) for reach cluster is also included in the cortical location labeling.

As shown in Figures 4, 5, the extracted sub-networks of SSM system are not exclusively localized to either side of the central sulcus rather its division is along the ventral-dorsal direction. This sub-division roughly separates the facial motor control from those for the rest of the body, as illustrated by the results of stimulus-evoked responses (Penfield and Boldrey, 1937; Mayka et al., 2006).

Another finding is that the cophenetic distance, defined as the threshold height at which the two sub-network groups join together, between two sub-network groups is not determined by the anatomical spatial distance rather than the level of functional conjunction between the involved brain sub-networks. For example, the cophenetic distance between auditory (Figure 4, clusters 1–3) and paracentral lobule (Figure 4 cluster 4) sub-network groups is 0.72, which is closer than that between auditory and facial-expression control (Figure 4, clusters 11–13) sub-network groups (0.74) or between auditory and hand control (Figure 4 clusters 6, 7, 9, and 10) sub-network groups (0.76).

The unilateral sub-network group in the right hemisphere (Figure 4, clusters 15, 16) involves two anterior regions of the parietal lobe: Precuneus and inferior parietal, which have closely related functions for visual-spatial abilities and motor coordination strategies (Snyder et al., 1997; Mattingley et al., 1998; Margulies et al., 2010). The unilateral sub-network group in the left hemisphere (Figure 4, clusters 17–18) is the most loosely connected components in SSM network. It consists of lateral premotor cortex (cluster 17), anterior insula (cluster 18), and caudal auditory cortex (cluster 19). This sub-network group plays an important role in the sensorimotor integration to mediate the categorization of incoming speech sounds through reciprocal auditory-to-motor and motor-to-auditory projections (Schroeder et al., 2001; Foxe et al., 2002; Alho et al., 2014).

### Sub-dendrogram of the visual RFN

**Figure 6:**
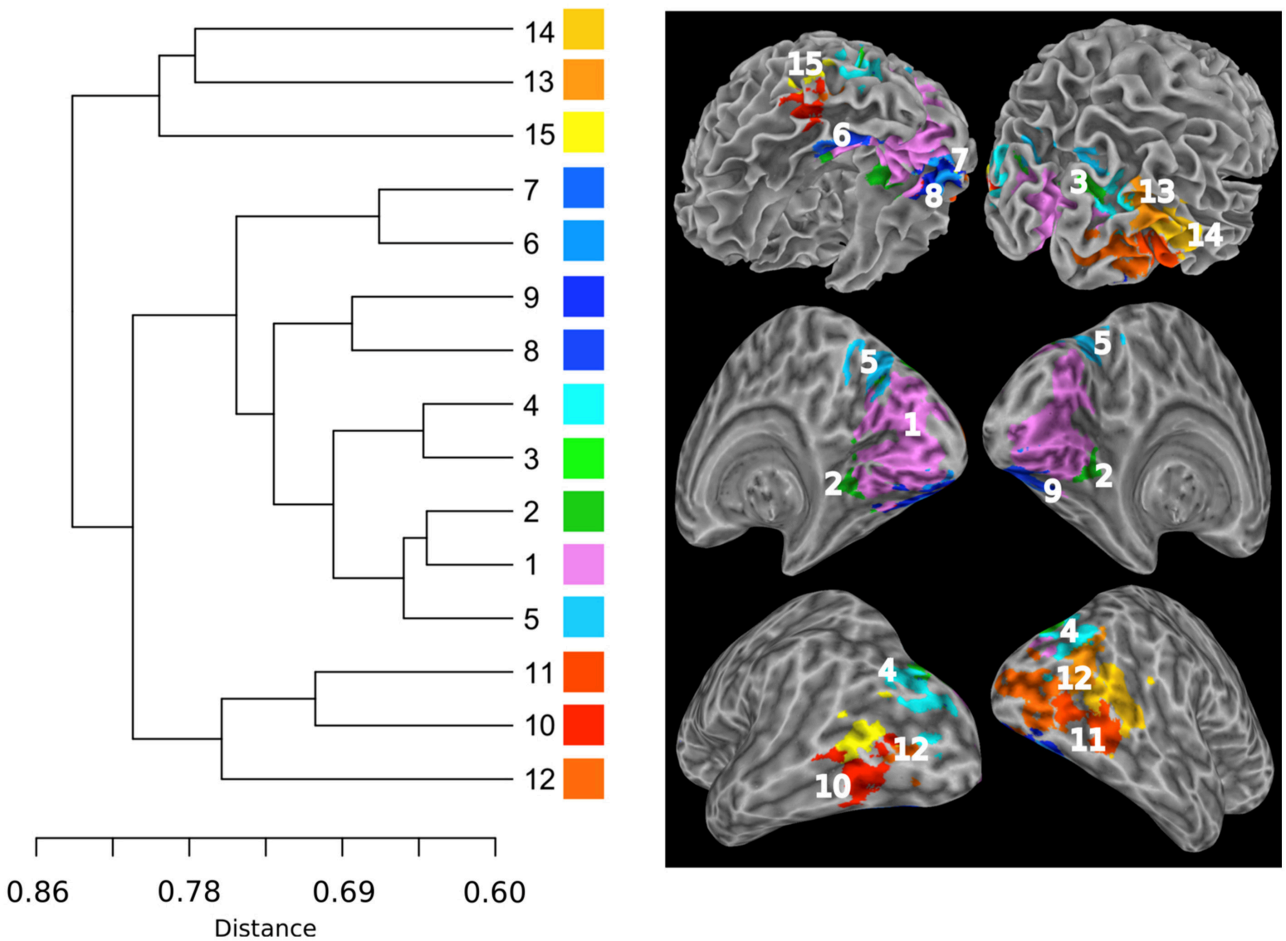
**Intra-network hierarchical organization of the sub-networks extracted from the visual system**. The lines in the dendrogram are drawn in proportion to the distance measure of the nodes (left). The sub-networks are labeled in ascending order according to their distances in the sub-dendrogram. Each sub-network is color coded to depict the neuroanatomical location in the Talairach template. The top panel shows the color-coded sub-networks imposed on the smoothed white matter surface. The middle and bottom panels show the medial and lateral views of the inflated hemispheres, respectively.

Figure 6 shows the hierarchical clustering results for the visual RFN at cutting level *k* = 79. Among the 80 nodes, 15 sub-networks amongst 14 dissociable nodes were identified (Tables 1, 3) to be statistically significant (*p* < 0.01 and *S* ≥ 12) and functional distinct. The intra-network hierarchical organization of the extracted sub-networks depicts five groups of bilateral sub-networks: primary visual cortex sub-network (Figure 6, cluster 1), ventral medial sub-network (Figure 6, cluster 2), dorsal sub-networks (Figure 6, clusters 3–5), ventral inferior-occipital sub-networks (Figure 6, clusters 6–9), and ventral posterior-temporal sub-networks (Figure 6, clusters 10–15).

**Table 3:** **Summary of the significant sub-networks extracted from the visual network**.

**Cluster**	**Size**	**Height**	**Cortical location**	**Focal point**
1	546	0.64	Cuneus; Lingual Gyrus;	(3, −74, 1)
			Striate and extrastriate Cortex; BA18, 30	
2	62	0.64	Left Lingual/Parahippocampal Gyrus;	(16, −49, −4)
			Right Lingual/Parahippocampal Gyrus; BA19	(−15, −53, −4)
3	37	0.65	Left Superior Cuneus/Precuneus;	(18 −81, 25)
			Right Superior Cuneus/Precuneus; BA18	(−18 −82, 24)
4	87	0.65	Left Middle Occipital Gyrus;	(−27, −83, 16)
			Right Middle Occipital Gyrus; BA19	(30, −77, 22)
5	86	0.66	Left Cuneus;	(14, −74, 29)
			Right Cuneus; BA18, 7, and 31	(−11, −78, 29)
6	150	0.67	Right Declive; Fusiform Gyrus; BA19	(4, −74, −20)
7	36	0.67	Left Declive: Fusiform Gyrus; BA19	(−32, −66, −22)
8	48	0.69	Left Fusiform Gyrus; BA19	(−23, −75, −14)
9	47	0.69	Right Fusiform Gyrus; BA19	(26, −74, −12)
10	33	0.71	Left Inferior Temporal Gyrus; BA19	(−41, −72, −1)
11	41	0.71	Right Inferior Temporal Gyrus; BA37	(45, −67, −2)
12	64	0.76	Left Middle Occipital Gyrus;	(32, −83, −1)
			Right Middle Occipital Gyrus; BA18	(−34, −82, 0)
13	45	0.77	Right Middle Temporal Gyrus; BA39	(40, −73, 19)
14	59	0.77	Right Middle Temporal Gyrus; BA37	(48, −61, 7)
15	33	0.80	Left Middle Temporal Gyrus; BA37	(−40, −73, 12)

The sub-networks are labeled in ascending order according to their heights in the hierarchical sub-dendrogram. The closest matching Brodmann area (BA) for each cluster is also included in the cortical location labeling.

Central to the visual network is the primary visual area sub-network (Figure 5, cluster 1). The location of cluster 1 overlaps with the anatomical location of the primary visual cortex V1 and dorsal visual association area V2d. These visual areas are strongly correlated with each other, as it is known that V2 receives strong feed-forward from V1 and sends feedback to V1 (Gazzaniga et al., 2002). The sub-network with the least cophenetic distance to the primary visual cortex is the ventral medial visual sub-network (Figure 6, cluster 2). The anatomical location of this cluster (Table 3, cluster 2) is consistent with the retinotopical locations of the ventral medial visual area, located within the posterior parahippocampal cortex (PHC) extending along the collateral sulcus and flanked by the lingual gyrus (Arcaro et al., 2009; Wang et al., 2015). This sub-network is known to respond more strongly to scenes than objects or faces (Arcaro et al., 2009).

Overall, the sub-dendrogram of the visual RFN reflects the known functional sub-division of the visual system summarized as the two-stream hypothesis that the ventral stream and the dorsal stream have distinct functional sub-divisions (Ungerleider, 1982). The dorsal visual areas are specific for the detection of motion, locating objects in space, and processing visual information used to guide movements (Goodale and Milner, 1992), whereas the ventral visual association areas are associated with form recognition and object representation (Vartanian and Skov, 2014). As shown in Figure 6, the cophenetic distance between the dorsal visual areas (Figure 6, clusters 3–5) and V1 (Figure 1, cluster 1) is 0.65, whereas the cophenetic distance between the ventral visual areas (Figure 6, clusters 6–15) and V1 is 0.72. Therefore, the primary visual cortex is more closely associated with the dorsal visual stream than that with the ventral visual stream.

The bilateral clusters 3 and 4 correspond to visual association areas V3a and V3b, respectively, both in terms of their anatomical locations and their lateral sub-divisions (Wang et al., 2015). V3a and V3b border the inferior parietal sulcus visual area (IPS0/1; Tootell et al., 1998; Press et al., 2001). This is consistent with our observation that clusters 3 and 4 are adjacent to the bilateral cluster 5, which corresponds to parietal visual areas IPS0. The cophenetic distance between the primary visual sub-network (Figure 6, cluster 1) and visual areas IPS0/1 (Figure 6, cluster 5) is 0.65, whereas the cophenetic distance between IPS0/1 and the visual association areas V3a and V3b (Figure 6, clusters 3, 4) is 0.69. This implies that IPS0/1 is more closely associated to the primary visual cortex than visual association areas V3a and V3b. This conforms the view that the parietal visual areas responsible for detection of motion, is comparatively faster relative to the ventral-temporal areas responsible for recognition and identification (Norman, 2002).

The ventral-stream visual association areas can be sub-divided into two sub-network groups according to the hierarchical functional sub-dendrogram: Inferior-occipital visual areas (Figure 6, clusters 6–9) and posterior-temporal visual areas (Figure 6, clusters 10–15). As shown in Figure 5, the inferior-occipital ventral visual areas are more closely associated to V1 with a mean cophenetic distance of 0.68, compared to the posterior-temporal ventral visual areas (Figure 6, clusters 10–15) with a mean cophenetic distance of 0.75. The anatomical location of cluster 6 overlaps with ventral visual area V2v and ventral visual area V3v (Wang et al., 2015). The clusters 7–9 forms bilateral clusters corresponding to ventral-temporal areas of human visual area V4 (hV4) and ventral- occipital visual area (VO1). Comparing with previously determined probabilistic maps of visual topography, we found that the boundaries of cluster 6 with clusters 7–9 are consistent with the known boundaries between hV4/VO1 and V3v (Sereno et al., 1995; DeYoe et al., 1996; Engel et al., 1997; Wade et al., 2002; Brewer et al., 2005). The clusters 10 and 11 correspond to the left and right middle-temporal visual area (MT)/V5 (Kolster et al., 2010), respectively. The bilateral cluster 12 corresponds well to the lateral occipital area 1 (LO1; Wang et al., 2015). The clusters 13–15 have the largest cophenetic distances to V1 in the visual network and correspond to the medial superior temporal area (MST; Wang et al., 2015).

### Variability of the HCA results based on averaged resting-state fMRI data

**Figure 7:**
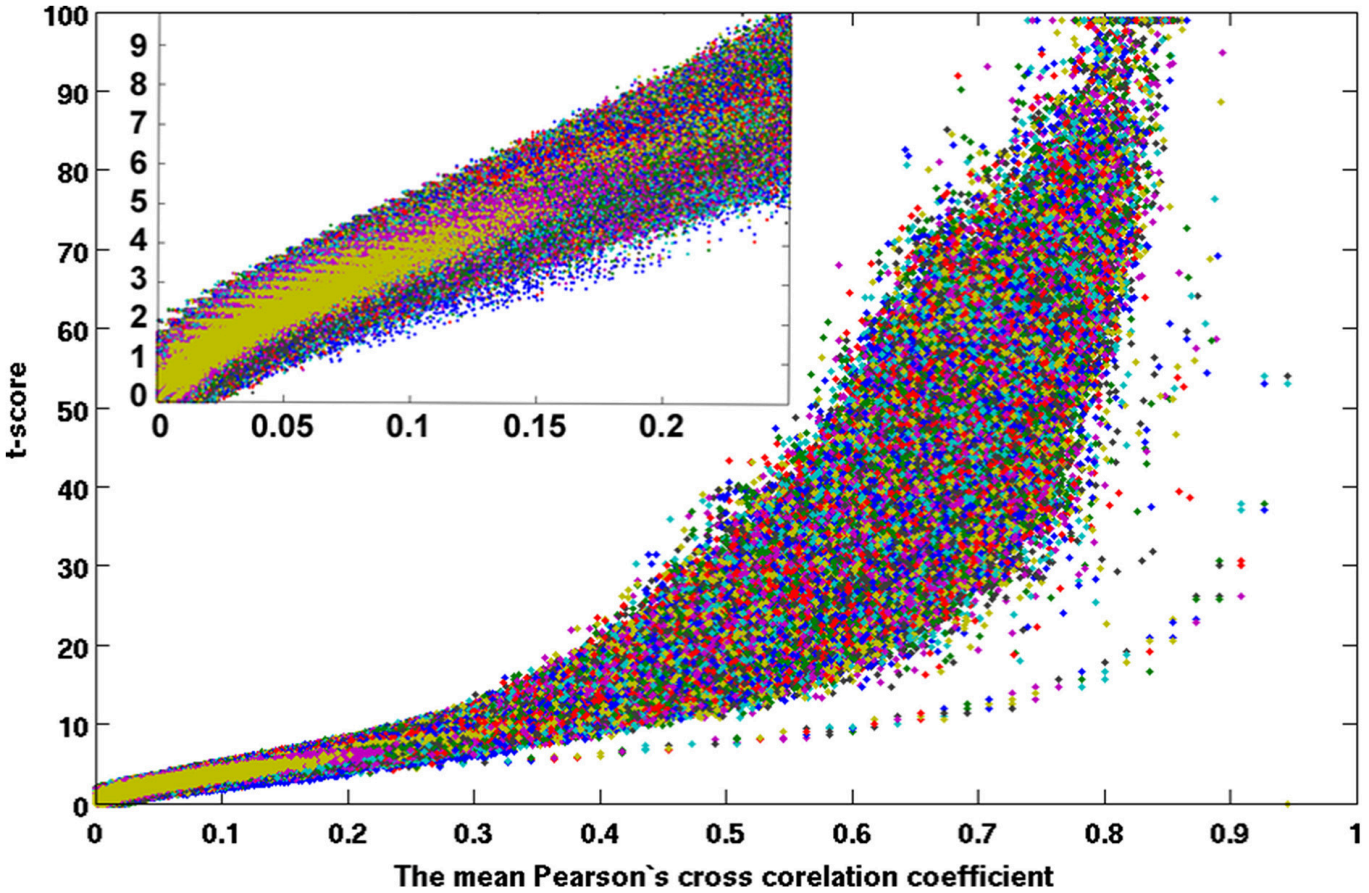
**A scattered plot of the one-sampled *t*-test score as a function of the cross-correlation coefficients of the resting-state fMRI time courses between voxels inside the brain**. The average results of 42 subjects (set 1) which were pseudo-randomly selected from the acquired whole dataset.

**Figure 8:**
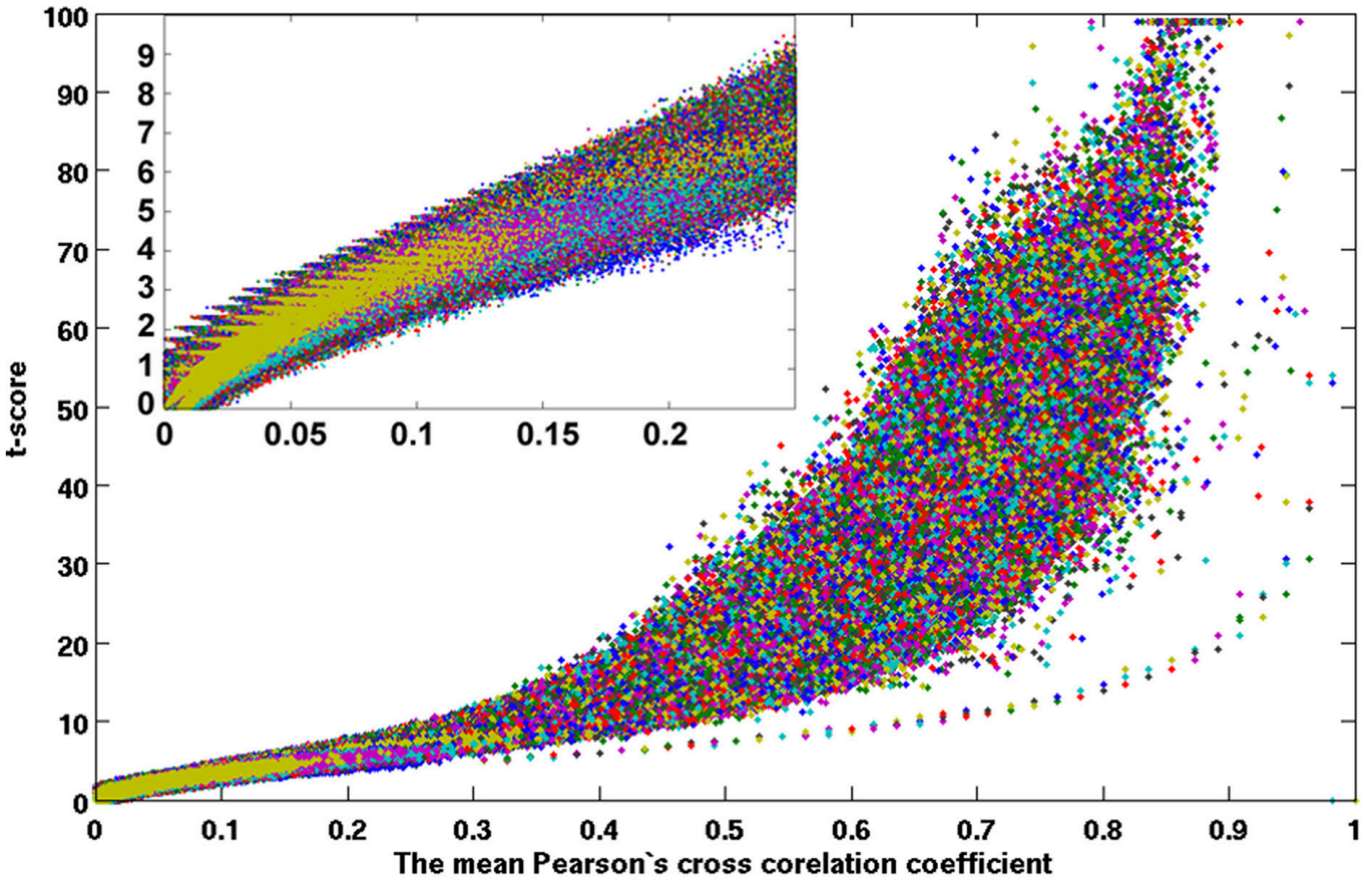
**A scattered plot of the one-sampled *t*-test score as a function of the cross-correlation coefficients of the resting-state fMRI time courses between voxels inside the brain**. The average results of 42 subjects (set 2) which were remaining half after excluding set 1.

**Figure 9:**
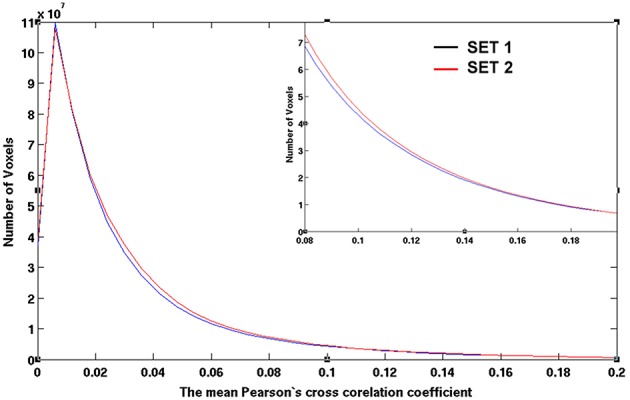
**The histograms of the averaged CC values for sub-datasets 1 and 2 as obtained by computing the number of voxels possessing CC values defined by an equally spaced bin interval of 0.006**. The CC values are the inter-subject average among 42 subjects after a CC ≥ 0.3 threshold at the individual level.

**Figure 10:**
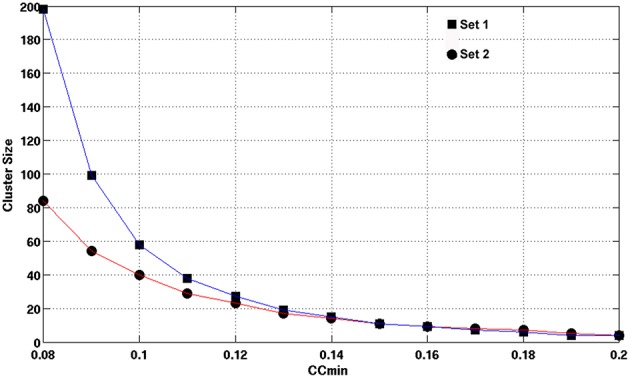
**The empirical cluster size criterion as a function of the minimum cluster CC values for datasets 1 and 2, respectively**. As assessed by Monte Carlo simulation with AlphaSim, the minimum cluster size guarantees a FWER, *p* ≤ 0.0.

Figures 7, 8 show the scattered plots of the voxel-wise *t*-score as a function of the voxel-wise CC of the average CC matrices for the two split sub-datasets, respectively. These scattered plots depict similar trend as for that of the entire dataset shown in Figure 2. The differences between the two datasets can be more clearly observed in the histograms of their average CC matrices and the corresponding criterion curves of the minimum cluster size shown in Figures 9, 10, respectively. It is clear that the derived HCA results are very sensitive to the CC variation in the interval 0.02 < CC < 0.12 where the cluster size criterion exhibits substantial difference between the two split sub-datasets.

## Discussion

### Voxel-wise hierarchical clustering of resting-state fMRI data

The agglomerative hierarchical clustering algorithm used in this study works by grouping the voxels one-by-one on the basis of the nearest distance measure defined by the pairwise correlation coefficient between the voxel data points. It can inherently stratify the image data into hierarchical structures of different scales, such as RFNs and sub-networks within a given RFN. We have previously used HCA to analyze whole-brain resting-state fMRI data voxel-by-voxel and obtained large-scale RFN results that are comparable with what were obtained from ICA studies (Wang and Li, 2013). One of the main issues in using HCA of resting-state fMRI data to extract large-scale RFNs is the prerequisite to specify the number of clusters produced by the algorithm. Neither the number of RFNs present in the data nor the noise characteristics are known *a priori* when applying the algorithm to a given resting-state fMRI dataset. In other words, we need to establish the cutting depth of the dendrogram. After testing the framework with the group-averaged resting-state fMRI data using different number of clusters and iterations to investigate how the choice of clustering parameters affect the outcomes, we proposed a multiple-iteration clustering scheme in combination with a cluster-size based criterion as the decision rules to extract potential RFNs (Wang and Li, 2013). It should be noted that the matching between HCA and ICA results is far from perfect, because these are two different data-driven techniques. The advantages of hierarchical clustering include data-driven characteristics and no specific assumption. ICA is also data-driven but makes strong assumptions about the data and underlying sources. The comparison was provided as a discussion to facilitate the readers to interpret the clustering results within the purview of previous RSN findings from ICA, but it was our expectation that clustering results must match precisely with prior RSNs.

In this study, we further explored the potential of voxel-based hierarchical clustering of resting-state fMRI data and extended the previously developed framework to extract sub-networks within SSM and visual RFNs. As discussed above, the intra-network hierarchical organizations of the intrinsic functional connectivity derived from spontaneous activity of the brain at rest reflect consistently the known functional modular organizations of the corresponding neural networks. It is apparent that the level of details that can be achieved using the voxel-wise HCA framework spans from the large-scale RFNs down to a single voxel defined the fMRI spatial resolution. For simplicity of comparison in this study, we chose the 1st crossing point of their IC curves as the clustering stop condition. With this choice we have also taken into consideration of that the number of clusters should be within a manageable magnitude.

There are limitations in the implementation suggested here. Firstly, a threshold of 2.65 for the IC curves corresponding to the first crossing point is arguably somewhat arbitrary. As shown in Figure 1, the IC curves do not reach a plateau until IC < 2.0, which corresponds to a cluster count over 200. This indicates that we can obtain substantially more clusters with considerable differences in correlation distance by lowering the threshold to IC > 2.0. However, further lowering the threshold gives rise to mostly smaller clusters, which are split-offs from the small clusters. It is difficult to give putative functional assignments to these split-offs. As shown in Tables 2, 3, most clusters extracted at the threshold of 2.65 have already become relatively small except for the core clusters (cluster 4 in SSM and cluster 1 in visual RFN). It is more productive to dissect selectively the largest cluster with an additional iteration. Secondly, the criterion for cluster size and weakest linkage (minimum mean CC) were not automatically combined with the IC threshold instead they were manually implemented in a stepwise fashion. Thirdly, the scope in exploring the relationship between cluster size and other cluster validity indices is so far quite limited. This aspect is particularly important when empirical knowledge for deriving cluster size criterion is unavailable, such as in the analysis of single subject data. Preliminary result indicates that the relationship between cluster size and the number of normalized connections is potentially useful for developing robust quality criterion (Wang and Li, 2013). Exploring the relationship between the cluster size and other commonly used cluster validity indices, such as, IC and root square mean of standard deviation can also be informative. In single subject-based clustering, several studies have reported that spatial reproducibility is a promising quality measure for resting-state fMRI data (Zhou et al., 2006; Chan et al., 2008; Ferrarini et al., 2009; Bellec et al., 2010; Alho et al., 2014; Russo et al., 2014; Mejia et al., 2015).

The implemented hierarchical clustering scheme is, nevertheless, a principled means in which certain internal characteristics of the data, such as cluster size, IC, and weakest linkage, functioned as a validity metric for selecting the clustering results. The obtained sub-dendrograms for the SSM and visual system corroborate remarkably well with the known modular organizations from previous clinical and experimental studies. On the other hand, not only the core sub-networks (cluster 4 for SSM and cluster 1 for the visual RFN) are sufficiently large to warrant further iterations, it is also very interesting to investigate further their functional and structural subdivisions. Result from preliminary testing (not shown) indicates indeed that an additional HCA iteration applied directly to these clusters can produce interesting subdivisions that can lead better understanding of the functional organizations within these sub-networks.

### Hierarchical modular organization of functional connectivity

The hierarchical modularity of brain functional network has been suggested to exist on multiple topological scales: Within each module there are a set of sub-modules, and within each sub-module a set of sub-sub-modules, etc. (Hilgetag et al., 2000; Zhou et al., 2006; Cohen et al., 2008; Ferrarini et al., 2009; Park and Friston, 2013; Zhen et al., 2013; Russo et al., 2014). At a given topological scale, a module is often made up of densely inter-connected, anatomically and/or functionally related cortical regions, while inter-modular connections tend to be relatively long-distance and sparse. Although the detailed organization of brain networks across all scales is currently not yet fully experimentally accessible, there is a rapidly growing arsenal of data analytic tools, including the voxel-based hierarchical clustering developed in this study, that have been tested to analyze complex dendrograms of brain structural and functional networks.

At the large-scale and global levels, our understanding of brain connectivity topology relies mainly on the analysis of anatomical and functional connections measured by non-invasive brain imaging (Hilgetag et al., 2000; Zhou et al., 2006; Cohen et al., 2008; Ferrarini et al., 2009; Park and Friston, 2013; Zhen et al., 2013; Russo et al., 2014). Both clustering (Golland et al., 2008; Wang and Li, 2013) and ICA of brain imaging data (Wang and Li, 2015) are particularly important data-driven approaches to study brain network organization. Accumulating results from clustering and ICA studies demonstrate that the cerebral cortex can be divided into the extrinsic and intrinsic systems at the global scale (Golland et al., 2008). The former comprises the sensory–motor cortex and is associated with the external environment. The later overlaps substantially with the default mode network and is likely associated with inner-oriented processing. The development of voxel-based hierarchical clustering of brain imaging data provides not only a complementary approach to ICA for the analysis of brain networks at large-scale level, it allows also the study of sub-dendrograms of a given network or even sub-network as demonstrated by the results from this study. It can be used to explore the sub-units of a brain network down to the level of voxel of a few millimeters limited ultimately by the spatial resolution of the *in vivo* brain imaging data acquisition techniques.

Not only empirical data confirm the hierarchical modular organization at different scales (Hilgetag et al., 2000; Park and Friston, 2013), but also theoretical considerations favor the assumed hierarchical modular topology of brain networks (Meunier et al., 2010; Bullmore and Sporns, 2012; Moretti and Muñoz, 2013; Park and Friston, 2013). It has been hypothesized that hierarchical modular organization of the brain is evolutionary advantageous, contributing to adaptive aspects of anatomical and functional brain connectivity. It enables the efficient processing of information, supports complex brain functions, and fits particularly for diverse dynamics and divergent functionalities within a fixed structure.

In this report we limited our exploration within the intra-network sub-dendrograms of the SSM and visual RFNs at rather crude scale. In the future we will explore for using the developed HCA framework to study the following topics: (1) extract sub-dendrograms for other RFNs such as the default-mode network; (2) refine sub-dendrograms of the large clusters of the SSM and visual RNFs based on resting-state fMRI data at higher spatial resolution. We believe it is feasible to use voxel-based hierarchical clustering of resting-state fMRI data for detailed retinotopical and somatosensory mappings; (3) study the variations in sub-dendrograms, particularly in subjects with neurological and neuropsychiatric diseases.

### Variability of the HCA results

The most direct method to study the variability of the HCA results is to compare the extracted dendrograms. However, it is not trivial to compare two dendrograms with excluded small clusters and leaf nodes. Since the employed HCA algorithm is completely deterministic, the extracted dendrogram results for the average resting-state fMRI data is, therefore, fully determined by the average CC matrix and applied validity criterion, which are also based on the CC matrix. It is apparent that the reproducibility of the HCA result is determined by the reproducibility of the CC matrix, which is affected by inter-individual differences in human brain function. The analysis of the variation in CC matrix discussed above provide an alternative approach to assess the variability of HCA result.

Any factor that can impact intra- or inter-individual variability of resting-state fMRI measurements can significantly influence the reproducibility of the average CC matrix. For example, age as a demographic factor, has recently has been demonstrated to have a significant influence on the test-retest reliabilities of functional connectivity (Song et al., 2012). Physiological noise originating from respiration and cardiac processes, impact resting-state fMRI signal, and are potentially detrimental to the reliability of the CC matrix (Murphy et al., 2013; Andellini et al., 2015). Involuntary head motion exhibits large inter-individual variability and can also have a large influence on the reliability of the CC matrix (Zuo et al., 2013). Furthermore, the dynamic nature of the resting-state functional connectivity implicates that variability of the CC matrix is not only inevitable but also unpredictable (Chiang et al., 2015). More detailed discussion on the physiological confounding factors influence the reliability of resting-state fMRI measurements have been discussed in recent reviews (Murphy et al., 2013; Andellini et al., 2015). Their impacts on the test-retest reliability of the CC matrix and derive HCA results require further systematic investigations, which is apparently beyond the scope of the current study.

## Conclusion

Using the SSM and visual RFNs as examples, we have demonstrated that the developed HCA framework is a useful tool for analyzing resting-state fMRI data voxel-by-voxel. Not only can it be used to extract the modular organizations of brain functional networks at the scale of large systems (Wang and Li, 2013), such as the entire SSM and visual systems, but also can it be used to derive the sub-dendrogram of a given RFN and dissect it at different level of details. The constructed sub-dendrogram for each RFN reveals the intrinsic functional connectivity among all sub-units within a RFN. For the two investigated RFNs (SSM and visual systems), the derived sub-dendrograms reflect consistently the known functional topographic mapping results. It should be possible to use the HCA framework for constructing sub-dendrograms of other RFNs and explore for potential but currently unknown functional junctions among the sub-units.

## Author contributions

YW was responsible for the design and implementation of the methods, as well as data analysis and manuscript drafting. MM handled interpretation and evaluation of results. TL initiated the study by providing overall design of the study and all data, also the main contributor of manuscript editing.

### Conflict of interest statement

The authors declare that the research was conducted in the absence of any commercial or financial relationships that could be construed as a potential conflict of interest.
